# Using geographic information systems and spatial and space-time scan statistics for a population-based risk analysis of the 2002 equine West Nile epidemic in six contiguous regions of Texas

**DOI:** 10.1186/1476-072X-6-42

**Published:** 2007-09-21

**Authors:** Min Lian, Ronald D Warner, James L Alexander, Kenneth R Dixon

**Affiliations:** 1 Division of Modeling and Geographic Information Systems, Institute of Environmental and Human Health, Texas Tech University/TTU Health Sciences Center, Box 41163; Lubbock, TX 79409, USA; 2 Department of Family and Community Medicine, Texas Tech University Health Sciences Center School of Medicine; Lubbock, TX 79430, USA; 3 Texas Department of State Health Services, Health Service Region 1, WTAMU Box 60968; Canyon, TX 79016, USA; 4 Department of Medicine, Washington University School of Medicine, Campus Box 8504, St. Louis, MO 63108, USA

## Abstract

**Background:**

In 2002, West Nile virus (WNV) first appeared in Texas. Surveillance data were retrospectively examined to explore the temporal and spatial characteristics of the Texas equine WNV epidemic in 2002. Using Geographic Information Systems (GIS) and the Spatial and Space-Time Scan (SaTScan) statistics, we analyzed 1421 of the reported equine WNV cases from six contiguous state Health Service Regions (HSRs), comprising 158 counties, in western, northern, central and eastern Texas.

**Results:**

Two primary epidemic peaks occurred in Epidemiological (Epi) week 35 (August 25 to 31) and Epi week 42 (October 13 to 19) of 2002 in the western and eastern part of the study area, respectively. The SaTScan statistics detected nine non-random spatio-temporal equine case aggregations (mini-outbreaks) and five unique high-risk areas imbedded within the overall epidemic.

**Conclusion:**

The 2002 Texas equine WNV epidemic occurred in a bi-modal pattern. Some "local hot spots" of the WNV epidemic developed in Texas. The use of GIS and SaTScan can be valuable tools in analyzing on-going surveillance data to identify high-risk areas and shifts in disease clustering within a large geographic area. Such techniques should become increasingly useful and important in future epidemics, as decisions must be made to effectively allocate limited resources.

## Background

West Nile virus (WNV) illness first was reported in the Western Hemisphere during the summer of 1999 from the New York City metropolitan area, where an epizootic appeared among particular avian species, horses, and humans. The human meningoencephalitis cases reported from New York City [1,2] initially were thought to be St. Louis encephalitis (SLE) virus infections [1]. However, concurrent illnesses/deaths of American crows, but not emus in the Bronx Zoo, and clinical disease/deaths in equines were not known to be attributable to the SLE virus [3,4]. Although WNV is an arbovirus that can be transmitted to humans and many other mammals, its natural cycle is mosquito-to-bird (Columbiform or Passerine amplifying reservoir species)-to-mosquito [5]. Mosquitoes become infected from the blood of a viremic bird and then, depending on the mosquito species, transmit the virus to non-reservoir birds, other vertebrate animals, and humans. An ongoing WNV epizootic has since progressed westward across the U.S., northward into Canada, and southward into the Caribbean, Mexico/Central America, and South America [6-10]. During this progressive epizootic, it has been documented that WNV also can be transmitted human-to-human via transplacental passage, organ transplantation, and transfusion of blood and related products [11], although these are considered minor routes. Animal-to-human or human-to-animal transmission has not been documented. Most non-avian vertebrate WNV infections are subclinical, but symptomatic infections can range in severity from an uncomplicated febrile illness to fatal encephalitis [4,11,12].

During the spring of 2002, Texas WNV illnesses were first detected in southeastern counties; avian fatalities, infected mosquitoes, and clinical disease among horses and humans were documented. Of all infected mammals, horses are among the most susceptible to clinical WNV disease. While most equines infected with WNV recover, nearly one-third of some reported equine cases have resulted in death or euthanasia [4,11,12]. Texas is "home" for nearly 15% of all U.S. horses [13]. According to the 2002 records of the Texas Department of State Health Services (TDSHS)[14] and the US Department of Agriculture [15], from a total of 372,341 horses and ponies, 1699 equine clinical cases were reported from 204 (80.3%) of Texas' 254 counties [16].

For an arthropod-borne disease, it is important to understand the spatial and temporal characteristics of its natural transmission. Adequate and effective analyses of this first equine WNV epidemic in a large area of a geographically diverse state, such as Texas, will assist in prevention and control efforts directed toward any future mosquito-borne epidemic. In this study, data from 1421 equine WNV case reports from six contiguous TDSHS Health Service Regions (HSRs) were analyzed using geographic information systems (GIS) and Spatial and Space-Time Scan (SaTScan) statistics to identify potential high-risk areas for WNV infection in these regions. Based on the previous general trend of east-to-west, state-to-state spread of WNV across the U.S., our preliminary hypotheses were: i) WNV entered southeast Texas, from the east via infected birds, in Spring of 2002 and subsequently spread westward (via infected birds) along rivers and their tributaries which drain the more-western regions; and ii) because of regional differences in equine vaccination status, climatic conditions, and mosquito-bird ecology, some areas of Texas would be at higher risk for equine WNV illness during the 2002 epidemic.

## Methods

### Data collection

**Figure 1:**
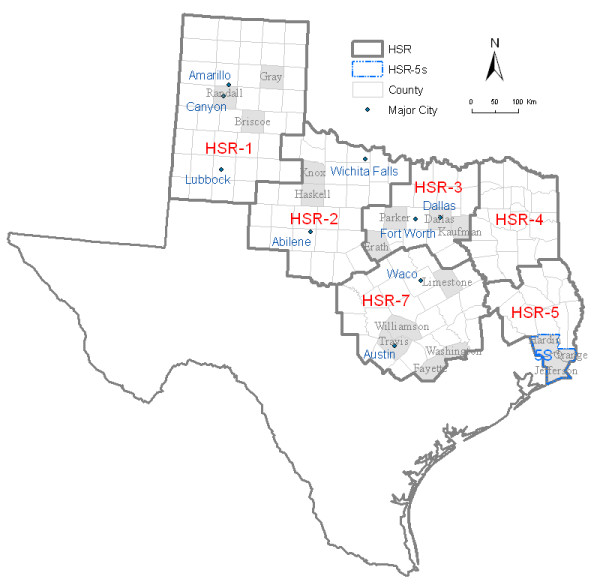
Study area for Equine WNV illness risk analyses: six contiguous Health Service Regions in Texas.

Data from all clinical equine WNV cases reported from six HSRs (see Figure 1) during 2002 were provided by TDSHS. HSR 1 is a 41-county area in the Panhandle and South Plains; HSR 2/3 is a contiguous 49-county area directly east of HSR 1; HSR 7 is the 30-county area south of HSR 2/3; and HSR 4/5 is a 38-county area east of HSRs 3 and 7. We did not use data from HSRs 6, 8–11 because these data lacked precise location information. Owners of affected equines were asked specific questions about reports of ill horses, including the physical location of each premise, date of illness onset, clinical symptoms (esp. any neurological signs, including encephalitis), and WNV vaccination history. Detailed information from the southern three counties of HSR 5 was not available and onset dates of these equine cases were estimated from TDSHS report dates [16]. Serum samples were collected by veterinarians and analyzed by the Texas Veterinary Medical Diagnostic Laboratory for serologic diagnosis, using an immunoglobulin M (IgM) antibody ELISA assay. In this study, a case was defined as a sero-positive equine for which an epidemiologic investigation form was completed. Based on this definition there were 1421 cases in 2002 from these regions (83.6% of all such reports in Texas during 2002).

### Data management and geocoding

The original regional datasets, provided as Microsoft Office Excel files, were imported to SAS^® ^(Version 9.1, SAS Institute Inc, Cary, NC) for data management and subsequent analyses. The address of the owner of each reported equine case was geocoded with longitude and latitude coordinates to "locate" the case, and then an equine case point-layer was developed, using the ArcInfo^® ^Geographic Information System (GIS, Version 8.3, ESRI, Redlands, CA.). The regional database was exported through the SAS System and joined to the equine case point-layer attribute table in ArcMap for further spatial analyses and mapping.

### Epidemic curves

Individual HSR and combined-HSRs cumulative epidemic curves were produced to illustrate the peaks and time trends of equine WNV onsets during the period June 27 through December 2, 2002.

### 3-Dimensional trend and space-time cluster detection, within and across regions

To illustrate the spatio-temporal relationships of the equine WNV cases in the study area, two steps were carried out based on the owner-reported onset date of each case combined with each equine case's spatial coordinates available in HSRs 1, 2/3, and 7. First, we produced a three-dimensional trend diagram with the longitude/latitude coordinates and the onset date for each equine case using Geostatistical Analyst Extension (ESRI, Redlands, CA.).

Then we used the Spatial and Space-Time Scan Statistics a cluster detection software application [17-19], using a retrospective space-time permutation model, to detect unique non-random space-time clusters. The SaTScan statistic has been used to detect potential geographic clusters of various human diseases [19-22]. This spatio-temporal statistical technique can detect probable location- and size-varied clusters in a defined geographic area [18,20,21,23]. A scanning window, circular (space-radius) or cylindrical (with an added time dimension), can detect probable geographic clusters as the "center" of the window moves over the study area. For each window centroid, the radius is continuously adjusted from zero to the user-predefined maximum. For the space-time permutation model, the time period is also adjusted from zero to the predefined maximum for each geographic circle. The Monte Carlo hypothesis method was used to test the statistical significance of possible clusters, and a *p *value was obtained by ranking the likelihood (random occurrence) of an observed cluster in the dataset over the maximum likelihoods of 999 randomly-produced datasets [17,24,25]. This method can adjust the multiple tests for different locations with varied sizes [25]. The original dataset was transformed to case and coordinate files for SaTScan cluster analysis. Based on the accepted incubation period of equine WNV disease, 14 days, and the approximate county size, 30 kilometer radius, the maximum cluster dimensions were set. The output from SaTScan was input into SAS to develop a cluster dataset which was then exported to ArcInfo GIS as a new geodatabase for mapping. A radius-range buffer layer was created to display significant cluster areas, based upon the cluster center point-layer. All clusters with *p *< 0.10 were mapped.

### Risk analysis: incidence distribution and geographic cluster detection

The equine WNV case database was organized to county-level case frequency data through the SAS System and joined to a county layer with the 2002 Texas equine census [15] as part of an attribute table in ArcMap, to produce a map displaying county-level incidence rates, using five cumulative periods illustrating the epidemic's temporal progression. To identify high-risk areas, we also performed a geographic cluster detection, estimating the equine population at risk using the Poisson model of SaTScan. Three types of data from each county were utilized for this analysis: the 2002 cumulative equine WNV case frequency; the 2002 equine census, and coordinates of the county's geographical centroid. The cumulative county-level case frequency was calculated for the five temporal periods from June 27 through: July 31; August 31; September 30; October 31; and December 2. These three data types for each county were imported to SaTScan for spatial cluster analysis. The maximum cluster size was set as 5% of the population at risk. We mapped all county-based temporal clusters if they yielded a *p *< 0.10.

## Results

### Epidemic curves

**Figure 2:**
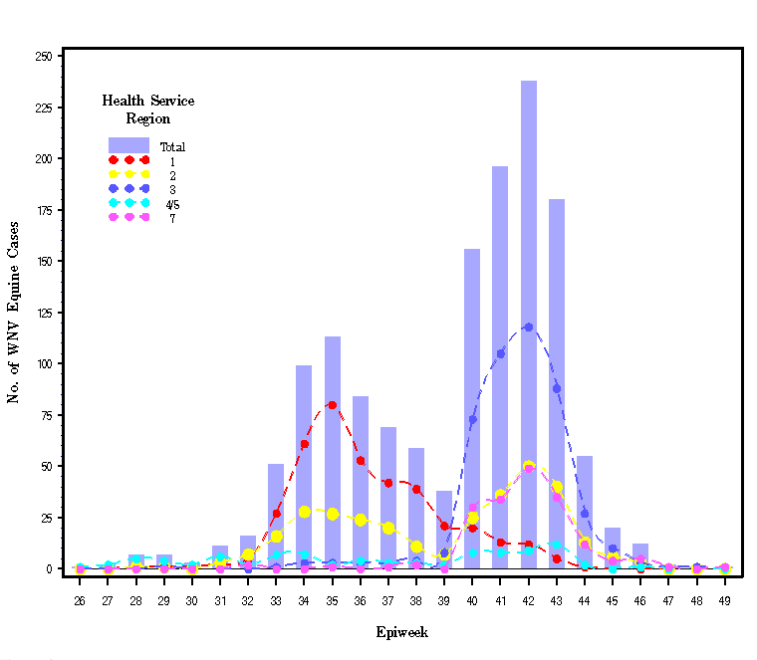
Epidemic curves of reported equine WNV cases from six contiguous Health Service Regions in Texas in 2002. Epiweek dates extend from Epiweek 26: June 23 to 29 through Epiweek 49: December 1 to December 7.

The overall 2002 equine WNV epidemic in Texas, which spanned 24 Epi weeks, produced two primary waves (temporal vectors) in these HSRs (Figure 2). The first wave, from Epi weeks 28 to 39 (primarily in HSRs 1 & 2), resulted in fewer cases than the second, from Epi weeks 39 to 47 (primarily all regions east of HSR 1). In HSR 1, case onsets began on July 7 and continued until October 29, peaking during Epi week 35 (August 25 to 31), and totaled 384 confirmed cases. In HSR 2, 318 equine WNV cases were reported, resulting in a bi-phasic outbreak curve during Epi weeks 32–39 and 40–45; case onsets ranged from July 13 until November 12. In HSR 3, 453 case onsets occurred during July 18 through November 26; this outbreak peaked during Epi week 42, seven weeks after the peak in HSR 1. In HSR 7, 176 equine WNV case onsets occurred from August 7 through December 2, peaking during Epi week 42. In HSRs 4/5, 90 cases occurred from June 27 through November 13 without a dramatic epidemic peak but, rather, a plateau from weeks 40 through 43.

### Space-time analyses

**Figure 3:**
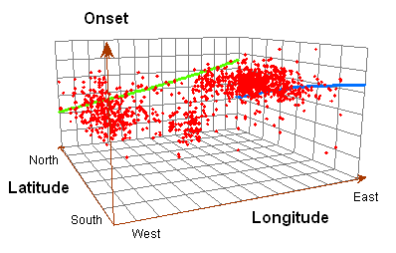
Three-dimensional spatio-temporal trends of the illness onset in equine WNV cases reported from four of six contiguous Health Service Regions (HSR 1, 2/3 and 7) in Texas in 2002. Green vector reflects the tendency of case onsets over longitude; blue vector reflects the tendency of case onsets over latitude.

Reported equine WNV case onsets tended to occur chronologically later from west-to-east, aside from the initial few cases in the southern portion of HSR 5 (Table 1), whereas onsets tended to occur earlier in higher latitudes (Figure 3). These data clearly suggest that onset dates of reported equine WNV illness were earlier in the northwest areas of Texas (HSR 1 and the western part of HSR 2) than in areas to the south and east (HSR 7, HSR 3 and the eastern part of HSR 2).

**Table 1:** The first five equine WNV cases, by date of onset and county, reported from six contiguous Health Service Regions in Texas; 2002. County locations are identified in Figure 1.

HSR	No.	County	Onset Date
1	1	Briscoe	Jul.7
	2	Randall	Jul.19
	3	Gray	Jul.25
	4	Randall	Aug.1
	5	Randall	Aug.2
			
2	1	Haskell	Jul.13
	2	Haskell	Jul.15
	3	Knox	Jul.31
	4	Knox	Aug.1
	5	Haskell	Aug.3
			
3	1	Parker	Jul.18
	2	Dallas	Aug.16
	3	Dallas	Aug.23
	4	Erath	Aug.23
	5	Kaufman	Aug.23
			
7	1	Fayette	Aug.7
	2	Washington	Aug.9
	3	Williamson	Aug.29
	4	Limestone	Sep.13
	5	Travis	Sep.16
			
4/5	1	Jefferson	Jun.27
	2	Jefferson	Jul.3
	3	Hardin	Jul.4
	4	Orange	Jul.7
	5	Orange	Jul.10

**Figure 4:**
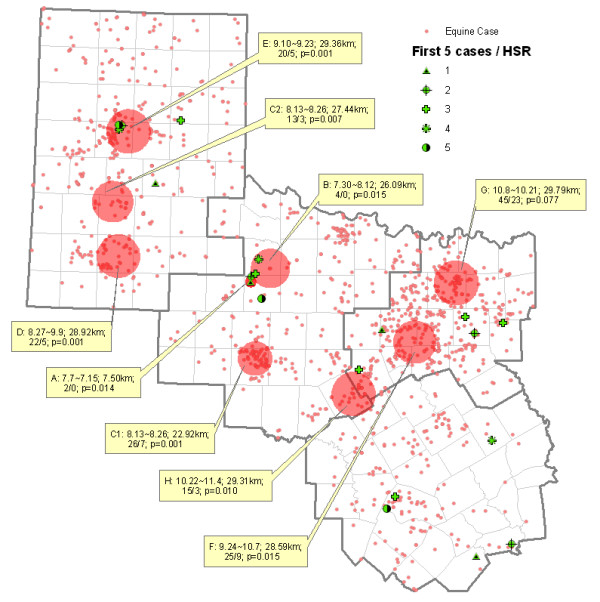
Space-time clusters of equine WNV cases reported from four of six contiguous Health Service Regions in Texas (HSR 1, 2/3 and 7) in 2002. Each cluster 'frame' provides time period of cluster (mm.dd), cluster radius, observed/expected cases, and p-value for rejecting the null hypothesis of no clustering.

Cluster analyses identified nine significant space-time clusters: three in HSR 1, three in HSR 2, two in HSR 3, and one where the boundaries of HSRs 2, 3 and 7 join (Figure 4). These clusters are time-serially coded (A – H), and statistical output is noted: the clustering period; radius; and observed/expected cases, with resulting *p *value.

### Risk analysis

**Figure 5:**
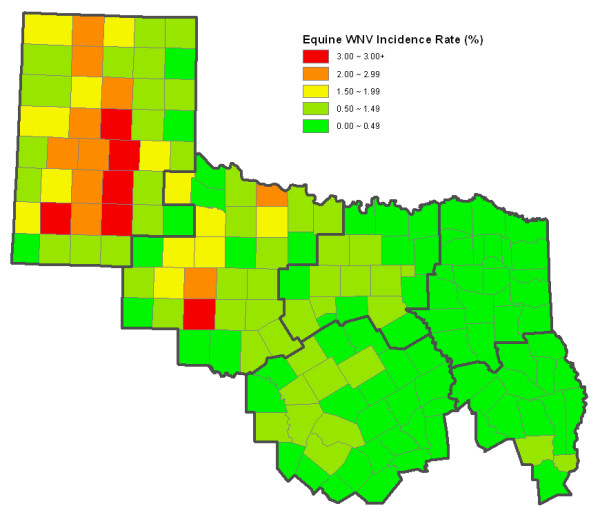
County-level cumulative incidence of equine WNV illness reported from six contiguous Health Service Regions in Texas in 2002.

The county-level cumulative incidence map (Figure 5) illustrates that the risk of equine WNV illness was the greatest in the south-central area of HSR 1 and in the south (Abilene area) and north (Wichita Falls area) of HSR 2. The incidence was relatively low in HSRs 3, 4, 5 and 7, although two counties in the southeast area of HSR 5 were at a higher-risk than the remainder of that region.

**Figure 6:**
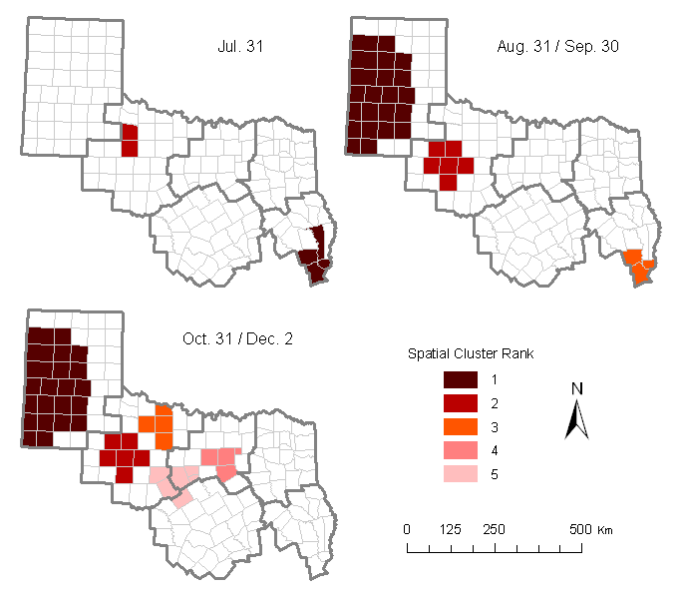
High-risk cumulative spatio-temporal clusters of equine WNV illness reported from six contiguous Health Service Regions in Texas in 2002; maximum cluster size was defined as 5% of the population at risk.

The monthly-cumulative spatial cluster analysis detected similar high-risk areas during different times (Figure 6 and Table 2). In July, the highest-risk areas were in the southern area of HSR 5 and the northwest part of HSR 2, contiguous with HSR 1. By August and September, the high-risk area in HSR 2 shifted more to the south and west of that region, and a new high-risk area appeared in more than half (22 counties) of HSR 1. By October, the third highest-risk area materialized in the Wichita Falls area, while the fourth and fifth highest-risk areas appeared in the Dallas/Ft. Worth area and the common boundary area of HSRs 2, 3 and 7; these remained stable through the end of the state-wide equine WNV epidemic of 2002.

**Table 2:** Significant spatio-temporal case-clusters in the 2002 Texas equine WNV epidemic in six contiguous Health Service Regions (See Figure 6 for cluster location).

Date (Until)	Cluster Rank	Observed cases	Expected cases	Log Likelihood Ratio	*p *value
Jul.31	1	19	1	59.52	0.001
	2	3	0	7.74	0.016
Aug.31	1	134	15	201.29	0.001
	2	54	5	80.45	0.001
	3	25	5	21.82	0.001
Sep.30	1	255	29	380.49	0.001
	2	95	10	133.35	0.001
	3	29	9	13.88	0.001
Oct.31	1	280	68	203.69	0.001
	2	113	24	89.85	0.001
	3	69	21	35.20	0.001
	4	96	53	14.98	0.001
	5	96	56	12.00	0.001
Dec.2	1	280	70	196.25	0.001
	2	113	25	86.98	0.001
	3	70	22	34.89	0.001
	4	104	54	18.75	0.001
	5	104	58	15.31	0.001

## Discussion

When WNV first appeared in Texas, the across-state "migration" routes of this virus were not completely understood. It was assumed that equines would likely experience more severe clinical disease from WNV infection than humans. During 2002, TDSHS documented 202 human cases of West Nile neuroinvasive disease (WNND), yielding a crude cumulative incidence of 0.93/100 K [26]; the 1699 equine cases yielded a crude cumulative incidence of 456.30/100 K [16]. Of the total 1699 reported equine cases, 1421 (83.6%) were analyzed in this study using GIS and SaTScan statistics.

Geographic Information Systems and SaTScan statistics comprise a very useful combination of methods to describe the progression of this epidemic and quantify unique spatio-temporal high-risk areas or outbreaks within the larger epidemic. A major advantage of this approach, in addition to more-completely characterizing epidemic durations and locations, is to provide a valuable early warning methodology by rapidly identifying increasing case counts and high-risk areas (both space-time permutation clusters and purely spatial clusters) utilizing routine ongoing surveillance data. The Minnesota Department of Health has applied this series of methods in syndromic surveillance [27].

Identification of significant disease clusters can provide valuable clues to an unknown or emerging disease etiology [19-22]. For infectious diseases, cluster analyses can play an important role in exploring the ecological variations and other relationships that support etiologic agents, e.g., between reservoir species, vector populations, and subsequent non-reservoir infections. In our study area, two distinct outbreak "waves" of an equine WNV epidemic were identified, along with previously unrecognized non-random clusters and movements or shifts in high-risk areas. One major outbreak began in mid-August and ended in mid-September, peaking in late August (HSRs 1 and 2); the other spanned October, peaking in mid-October (HSRs 2, 3, and 7).

The geographic analyses clearly demonstrate the time course of the 2002 equine WNV outbreaks within these regions of Texas. The cases occurred significantly earlier both in HSR 1 and the western half of HSR 2 than in the eastern half of HSR 2 and HSR 3. Additionally, the county-level cumulative incidence demonstrates an obvious geographic "risk gap" between the central portion of HSR 1 and the western portion of HSR 2. This suggests, in combination with simultaneous outbreak waves (Epi weeks 32 to 39) in both regions, that these western Texas outbreaks probably consisted of independent events in these areas during August 04 through September 28. This finding also suggests that the spread of WNV into HSR 1 and the western part of HSR2 may have come from the north/northeast (Kansas and/or Oklahoma), not from the southeast via HSR 3. This result is contrary to our preliminary hypothesis that WNV moved progressively westward through three of the more eastern HSRs. The temporal southerly and eastward shift of spatial high-risk areas within HSR 2 and into HSRs 3 and 7 provides additional evidence to support this conclusion. For the eastern part of HSR 2 and the whole of HSRs 3 and 7, that outbreak may have been a "mixed" event, with three possible "migration" routes: i) following earlier activity in HSRs 1 and 2, WNV may have been transmitted from the north/northeast to these areas at a lower velocity; ii) since there was an obvious bi-phasic character to the epidemic curve of the entire epidemic, WNV in the eastern part of HSR 2 and the whole of HSRs 3 and 7 may have spread from the western portion of HSR 2; and/or iii) spread from the first five cases (Table 1) and spatial cluster area in the southern portion of HSR 5, suggesting that WNV may have spread from the east, across HSRs 4 and 5 into HSRs 3 and 7. Such a "mixed" event is supported by Ward's study in which a kriging map showed that Texas equine encephalomyelitis case onsets developed in a two-point-derived mode [28].

The lower incidence of equine WNV illness in eastern and north-central Texas (HSRs 3–5 and 7) could have resulted from more and/or earlier WNV vaccination in these regions in 2002. Or, these lower regional incidence rates could reflect the proportion(s) of resident vector mosquitoes, with different efficiencies for transmitting WNV and/or differences in proportions of mosquitoes positive for WNV. In 2003 Texas mosquito surveillance, *Culex tarsalis *predominated (82.9% of pools) in HSR 1, while *Cx. quinquefasciatus *predominated (93.9–98.4% of pools) in HSRs 4/5 and 7; HSR 2 is a 'transition zone' (78.7% of pools were *Cx. quinquefasciatus*) between the other distributions; p ≤ 0.001 [26].

Although the TDSHS was very thorough in investigating every reported equine case during 2002, and owners/veterinarians were provided instructions on surveillance criteria, undoubtedly some cases were not reported. The extent of such underreporting is unknown, but we believe it is a small proportion and, most importantly, uniform across the HSRs in this study. In addition, a small number of confirmed cases (four, four, one, and seven from HSRs 1, 3, 4/5, and 7, respectively) were omitted from the analyses because of insufficient information: location, onset date, and/or diagnosis. There was an equine vaccine available before the 2002 WNV epidemic began in Texas. Based on anecdotal reports from veterinarians, more vaccine was sold and/or administered in the eastern regions of our study area, whereas very little vaccine was administered in HSR 1. Vaccination of some equines prior to or during the WNV epidemic could account for some of the lower cumulative rates, but would not impact the temporal findings of this study. In support of these findings, the age-adjusted relative risk of WNND in humans living in HSR 1 was 1.3 in 2002, and increased to 8.0 in 2003, compared to those residing in all other HSRs [26]. On large acreages of some ranches, as in some Texas counties, the longitude and latitude of an equine owner's address may not be the exact coordinates where the animal was exposed/infected. It is very likely, however, to be within the same county. And the majority of equine cases would likely not have traveled outside their county within the two-week WNV incubation period.

As an emerging infectious disease, now considered endemic by many, WNV has become an important concern of public health professionals in the U.S. It is very important, for epidemic control and prevention measures, to understand risk analyses and, thereby, identify case clusters and high-risk areas. Geographic information systems and SaTScan statistics provide very effective tools for these purposes. Our cluster analysis detected nine non-random space-time aggregations of equine cases, and six unique high-risk areas within the 2002 Texas epidemic. This study demonstrated that the high-risk areas we identified were spatially related to the high-risk areas of Texas during the 2003 human WNND epidemic [26]. With improvements in analytic techniques and access to disease surveillance data, it is becoming ever more important to effectively use those data in managing ongoing outbreaks and larger epidemics. Spatial epidemiology and GIS are playing more important roles in disease control and prevention. Our study suggests that population-based spatial and temporal analyses of initial surveillance data would be very helpful in managing the next vector-borne epidemic, by highlighting when and where limited public health or veterinary preventive medical resources should be concentrated.

## Conclusion

This study indicates that the 2002 equine WNV epidemic in Texas occurred in a bi-modal pattern, and WNV possibly "migrated" from the north/northeast (Kansas and/or Oklahoma) to northwestern Texas and from the southeast (Louisiana and/or Arkansas) to eastern Texas. Although the spatial and temporal analyses demonstrated significant temporal trends of equine WNV incidence, additional study is required to determine the definitive routes of WNV into Texas. The main findings of this study are: i) the 2002 equine epidemic did not spread uniformly across contiguous regions of Texas; and ii) even within the component regional outbreaks of the larger epidemic, "local hot spots" developed, i.e., statistically significant (non-random) spatio-temporal clustering of cases. This implies that GIS and SaTScan statistics can be effective tools to assist in the prevention and control of future epidemics by indicating where and when limited resources can be used most effectively.

## Abbreviations

GIS: Geographic Information Systems;

HSR: Health Service Region;

TDSHS: Texas Department of State Health Services;

WNV: West Nile virus.

## Authors' contributions

All four authors closely worked together to design this study, analyze the data, interpret the results, and finish writing this manuscript.
